# Drug Screening and Validation Targeting TDP-43 Proteinopathy for Amyotrophic Lateral Sclerosis

**DOI:** 10.14336/AD.2024.0440

**Published:** 2024-02-01

**Authors:** Jiaqi Xin, Sen Huang, Jing Wen, Yunhao Li, Ang Li, Senthil Kumaran Satyanarayanan, Xiaoli Yao, Huanxing Su

**Affiliations:** ^1^State Key Laboratory of Quality Research in Chinese Medicine, Institute of Chinese Medical Sciences, University of Macau, Macao, China.; ^2^Department of Neurology, The First Affiliated Hospital, Sun Yat-sen University; Guangdong Provincial Key Laboratory of Diagnosis and Treatment of Major Neurological Diseases; National Key Clinical Department and Key Discipline of Neurology, Guangzhou, China.; ^3^Centre for Regenerative Medicine and Health, Hong Kong Institute of Science & Innovation, Chinese Academy of Sciences, Hong Kong Science Park, Hong Kong, China.

**Keywords:** Amyotrophic lateral sclerosis, TDP-43 proteinopathy, Drug screen, Disease model

## Abstract

Amyotrophic lateral sclerosis (ALS) stands as a rare, yet severely debilitating disorder marked by the deterioration of motor neurons (MNs) within the brain and spinal cord, which is accompanied by degenerated corticobulbar/corticospinal tracts and denervation in skeletal muscles. Despite ongoing research efforts, ALS remains incurable, attributed to its intricate pathogenic mechanisms. A notable feature in the pathology of ALS is the prevalence of TAR DNA-binding protein 43 (TDP-43) proteinopathy, detected in approximately 97% of ALS cases, underscoring its significance in the disease's progression. As a result, strategies targeting the aberrant TDP-43 protein have garnered attention as a potential avenue for ALS therapy. This review delves into the existing drug screening systems aimed at TDP-43 proteinopathy and the models employed for drug efficacy validation. It also explores the hurdles encountered in the quest to develop potent medications against TDP-43 proteinopathy, offering insights into the intricacies of drug discovery and development for ALS. Through this comprehensive analysis, the review sheds light on the critical aspects of identifying and advancing therapeutic solutions for ALS.

## 1. Introduction

Amyotrophic lateral sclerosis (ALS) is an age-related neurodegenerative disease (NDD), marked by the accelerated decline of upper and lower motor neurons (MNs), leading to fatal paralysis within a typical progression timeframe of 2 to 5 years [1]. Globally, ALS is diagnosed at a rate of approximately 2 cases per 100,000 individuals annually, with incidence rates differing between sexes [2]. The general adjusted ratio of males to females with ALS is 1:3.5, highlighting a distinct gender-based disparity in its occurrence [3]. Around 10-15% of individuals with ALS have a familial history of the condition, defined as familial ALS patients (fALS), linked to pathogenic mutations in over 40 genes, including notable ones like chromosome 9 open reading frame 72 (*C9orf72*), superoxide dismutase 1 (*SOD1*), trans-activation response DNA-binding protein of 43 kDa (*TARDBP*), and fused in sarcoma (*FUS*) [4-8]. Conversely, approximately 85-90% of cases are sporadic ALS patients (sALS), where the genetic underpinnings remain elusive [9]. Most experts concur that the origin of ALS is rooted in a combination of genetic and environmental factors. Nonetheless, a segment of the research community champions more nuanced theories, such as the gene-time-environment interaction model and the multistep "hits" hypothesis, which propose a layered and intricate interaction of variables contributing to the disease's development [10-12]. The intricate pathogenesis of ALS presents substantial challenges in its treatment landscape. To date, no pharmacological intervention is capable of arresting or reversing the disease's trajectory. The treatments approved by the U.S. Food and Drug Administration (FDA), including Riluzole [13], Edaravone [14] and AMX0035 [15], demonstrate limited efficacy, marginally decelerating disease progression for a brief period. ALS distinguishes itself among neurodegenerative disorders with the approval of gene-modification therapies, highlighting the acute need for more effective clinical treatments for this rapidly progressing terminal condition.

In the quest to develop treatments that curb the progression of ALS, contemporary research primarily focuses on strategies that address initial causal factors, such as genetic mutations, and on creating pharmaceuticals that target and mitigate various aspects of the disease's complex pathophysiology. Gene therapy is advancing swiftly as a promising approach for ALS, mainly targeting genes directly linked to the disease's pathology. This innovative strategy is fostering clinical trials focused on ALS patients with specific genetic aberrations, including *SOD1* mutations [16], *C9orf72* hexanucleotide repeat expansions [17], *ATXN2* trinucleotide expansions [18], and *FUS* mutations [19]. Despite its promise, gene therapy for ALS faces significant hurdles, including its applicability to only a small subset of patients and the potential for off-target effects. Thus, the common pathophysiological alterations observed during the onset and advancement of ALS may provide viable targets for therapeutic intervention. *TARDBP* has been defined as the pathogenic gene of ALS. The proteinopathy of TDP-43 encoded by *TARDBP* has been observed in more than 97% of ALS patients [6, 20-22], suggesting that TDP-43 plays a crucial role in the pathophysiology of ALS and may be related to the occurrence and development of ALS. Therefore, its function and related pathology in ALS MNs have been extensively investigated.

**Figure 1.**
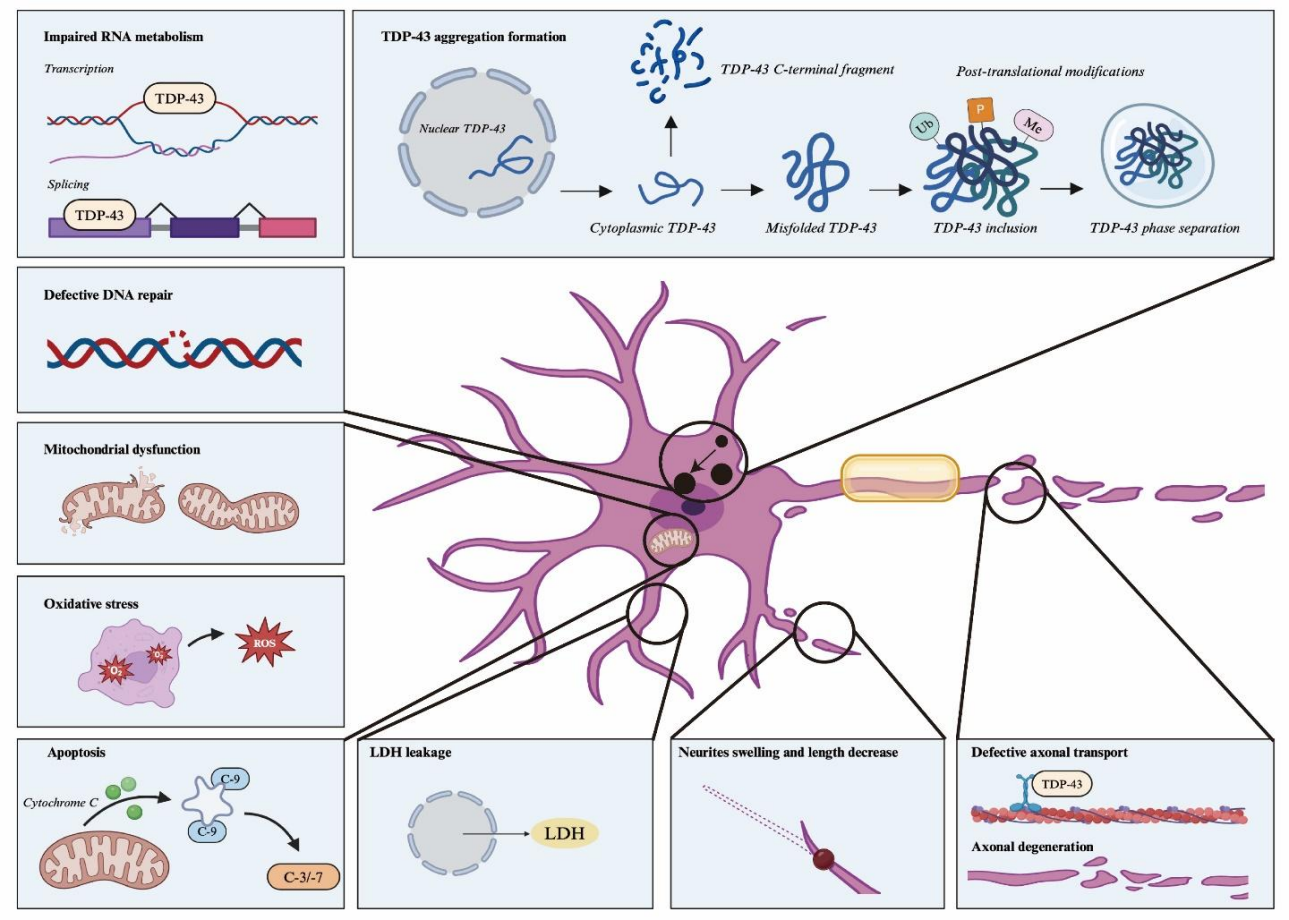
**Pathological phenotypes via dysregulated TDP-43 in ALS MNs**. Under normal physiological conditions, TDP-43 shuttles between the nucleus and cytoplasm to perform a variety of physiological functions. In ALS, the normal physiological function of TDP-43 is disrupted, resulting in multiple pathological manifestations, demonstrating neurotoxicity that may eventually lead to the death of MNs.

TDP-43, encoded by the *TARDBP* gene located at 1p36.22 on chromosome 1, is a critical element of the tau-negative, ubiquitin-positive inclusions that are hallmark features of both ALS and Frontotemporal lobar degeneration (FTLD) associated with TDP-43 pathology [23]. TDP-43 is a member of the heterogeneous ribonucleoproteins (hnRNPs) family, which are crucial for RNA processing in eukaryotic cells. It comprises an N-terminal domain (NTD), two consecutive RNA recognition motifs (RRM1 and RRM2) that bind nucleic acids, and a C-terminal domain. This latter domain includes a prion-like intrinsically disordered region (IDR) that facilitates TDP-43's liquid-liquid phase separation (LLPS). Notably, this region harbors most ALS-linked mutations, such as A315T, M337V, A382T, and Q331K, among others [24, 25]. Under normal physiological conditions, TDP-43 is primarily localized in the nucleus, although a fraction continuously shuttles between the nucleus and the cytoplasm. In these regions, it performs a variety of critical biochemical roles, including RNA processing, mRNA stabilization, trafficking, and miRNA processing [26-28]. Additionally, TDP-43 is involved in translation, the dynamics of stress granules (SGs), and the regulation of mRNA stabilization [26-28]. However, in the context of disease, TDP-43 predominantly accumulates in the cytoplasm, where it experiences misfolding and atypical liquid-liquid phase separation (LLPS). A series of aberrant post-translational modifications accompany this, including hyper-phosphorylation, ubiquitination, sumoylation, methylation, acetylation, and nitrosylation [29-32]. Additionally, fragmentation of the C-terminus, primarily into TDP-35 and TDP-25 fragments, occurs further facilitating the aggregation and deposition of the protein (Fig. 1). The simultaneous depletion of TDP-43 from the nucleus and its accumulation in the cytoplasm has led researchers to propose that TDP-43 contributes to neuronal death and the advancement of ALS through mechanisms of loss-of-function (LOF), gain-of-function (GOF), or a combination of both. Previous studies using conditional and partial knockout models have shown that a reduction in TDP-43 function can lead to motor neuron defects, a characteristic symptom of ALS [33, 34]. Emerging evidence suggests that loss-of-function mechanisms in ALS may arise due to the sequestration of soluble TDP-43 within cytoplasmic aggregates, rendering it incapable of performing its standard cellular roles. These functions span a broad spectrum, from preventing DNA damage to managing various facets of RNA processing, including transport and translation in the cytoplasm, as well as correcting mRNA splicing aberrations [35-42]. Recent findings indicated that the nuclear loss-of-function (LOF) of TDP-43 significantly impairs the regulation of pre-mRNA splicing, particularly affecting genes such as stathmin-2 (STMN2) [43-45] and UNC13A [46, 47]. This disruption leads to the uncontrolled inclusion of cryptic exons and compromises the stability of hundreds to thousands of RNA transcripts in both a species-specific and cell-specific manner [20, 48-50], closely linked to axonal and synaptic disruptions, exacerbating the pathological landscape of the disease.

The accumulation and aggregation of TDP-43 in the cytoplasm are associated with various toxic GOFs. Experiments have shown that overexpression of wild-type TDP-43 (WT TDP-43) can lead to TDP-43 pathology in MNs in a variety of ALS animal models, suggesting that GOFs of TDP-43 may be one of pathogenic mechanisms [51-53]. The GOF mechanisms include the direct toxicity of TDP-43 aggregates, the harmful effects of C-terminal fragments, and the indirect toxicity resulting from the entrapment of proteins normally interacting with TDP-43 within these aggregates. For instance, an excess of TDP-43 might lead to the formation of dysfunctional complexes as the binding partner proteins [54-56] become scarce, overloading other cellular organelles, and resulting in their functional decline.

Subsequently, the LOF and GOF further cause the imbalance of mitochondrial homeostasis, increasing oxidative stress, axonal transport defects and widespread inflammatory response. Finally, a feed-forward loop is formed to aggravate the progression of ALS [57]. Therefore, reestablishing TDP-43 homeostasis emerges as a promising strategy for ALS treatment. Strategies to achieve this can be categorized into three primary objectives: firstly, the indirect adjustment of TDP-43 expression through methods such as knockdown or overexpression; secondly, the modulation of TDP-43 levels *via* the enhancement of protein clearance pathways, including the ubiquitin-proteasome system (UPS) or autophagy; and thirdly, ensuring proper intracellular localization of TDP-43 and preventing the formation of its pathogenic aggregates. A critical initial step involves developing precise screening models to identify potent compounds that operate effectively across these strategies for ALS treatment.

A variety of drug screening models targeting TDP-43 proteinopathy have been developed, focusing on specific aspects of the pathology, such as modulating TDP-43 expression levels [58-60], correcting cytoplasmic mislocalization [61, 62], altering post-translational modifications [63, 64], influencing SG dynamics [65], and enhancing clearance mechanisms [66-69] in both *in vitro* and *in vivo* settings. These validation models not only replicate key pathological features like motor neuron death, TDP-43 abnormalities, and glial cell activation but also exhibit a range of ALS-related phenotypes, including muscle atrophy, dyskinesia, and reduced lifespan [70-73]. These comprehensive disease models lay the groundwork for developing ALS therapies aimed at TDP-43 proteinopathy. Thus, it is intriguing to consider how the strengths and weaknesses of these models can be leveraged to advance drug development efforts.

Effective preclinical screening cascades for drug discovery are underpinned by disease models that bolster confidence in the potential clinical translation of preclinical findings. This is particularly crucial for NDD, where the challenge lies in identifying models that accurately predict clinical outcomes. There is a pressing need to refine ALS disease models to enhance their utility as platforms for drug development. Currently, both the screening and validation models for TDP-43 proteinopathy lack standardization and comprehensive evaluation. Furthermore, the rationale behind the development of each model, along with their respective strengths and weaknesses, has not been systematically reviewed. In this review, we provide a detailed summary of the key drug screening and validation models targeting TDP-43 proteinopathy, illustrate notable examples of drug development, and critique the advantages and disadvantages of these models. Our goal is to offer insights and resources that could support the advancement of clinical treatments for ALS.

## 2. Drug screening models targeting TDP-43 proteinopathy

Drug screening models are critical experimental techniques utilized to demonstrate the pharmacological effects of specific substances. These methodologies are essential for the identification and discovery of new therapeutic agents. Utilizing a range of high-content and high-throughput screening approaches specifically designed to target TDP-43 proteinopathy, researchers are now exploring potential compounds from extensive libraries, including tens of thousands of natural products, small molecular entities, and FDA-approved drugs. In this context, we provide a comprehensive overview of both *in vivo* and *in vitro* drug screening models focused on TDP-43 proteinopathy.

### 2.1 *In vitro* drug screening models

#### 2.1.1 Targeting TDP-43 expression levels

To mitigate the GOF toxicity associated with TDP-43, initial research efforts focused on reducing the levels of TDP-43 protein itself as a therapeutic strategy. These studies employed the InCell Western (ICW) assay for drug screening, which utilizes TDP-43 specific antibodies at various dilutions. This technique quantifies the fluorescence intensity recorded by the Odyssey Infrared Imaging System (LI-COR) to assess the depletion of TDP-43 by potential therapeutic compounds in N9 cells. After screening 281 compounds, researchers discovered a novel compound, hexachlorophene (also referred to as B10), which was found to significantly reduce TDP-43 levels [74].

#### 2.1.2 Targeting TDP-43 aggregates formation

The assembly of TDP-43 may be a critical precursor to its eventual aggregation. Consequently, research has increasingly focused on disrupting the transformation of soluble TDP-43 monomers into insoluble inclusions. Compounds that modulate TDP-43 self-interaction could potentially interrupt the formation of pathogenic oligomeric aggregates, offering therapeutic potential. To this end, Moritz et al. developed a NanoBit luciferase complementation assay that quantifies TDP-43 self-interactions [75]. Utilizing this assay, they conducted a screening that led to the identification of auranofin, chelerythrine, and riluzole as dose-dependent inhibitors of TDP-43 self-interaction, importantly, without diminishing TDP-43 expression.

The interaction between TDP-43 and stress granule (SG) biology in TDP-43 proteinopathy opens novel pathways for addressing TDP-43-linked pathological processes. SGs form temporarily as a survival strategy in response to cellular stress, serving as an adaptive mechanism [76]. Recent research reveals that TDP-43 is recruited to SGs in various cell types under conditions such as heat shock [77], oxidative stress [78], and chemical stress inducers [79]. Dewey et al. developed a sorbitol-induced model of TDP-43 pathology in HEK293T cells and primary cultured glial cells, demonstrating differential stress responses between cells expressing WT and mutant TDP-43. Notably, mutant TDP-43 was incorporated into stress particles more rapidly and formed substantially larger SGs than its WT counterpart [79]. Boyd et al. developed a cellular high-throughput screening model to explore the pathophysiological effects of TDP-43 aggregation. Rather than directly disrupting protein aggregation pharmacologically, this model aimed to alter the process of SG formation [80]. They screened a chemical library containing 75,000 compounds and identified 16 compounds that could reduce TDP-43 inclusions in a dose-dependent manner, with further validations conducted in cell and nematode models. Additionally, Fang et al. designed a drug screening system that targets the formation of G3BP1-positive SGs in HEK293T cells and neural progenitor cells (NPCs) under oxidative stress induced by sodium arsenite (NaAsO2). This system pinpointed molecules with planar structures capable of preventing the localization of TDP-43 to SGs and diminishing its accumulation in cytoplasmic puncta in mutant induced pluripotent stem cell-derived MNs (iPSC-MNs) [81].

As a member of the heterogeneous nuclear ribonucleoprotein (hnRNP) family, TDP-43’s transport and accumulation are influenced by specific kinases, notably within the mitogen-activated protein kinase (MAPK) pathway [82]. Building on this, Moujalled et al. employed an *in vitro* model for TDP-43 positive SG formation to investigate the impact of kinase inhibitors [83]. Surprisingly, while some inhibitors, especially those targeting the MAPK pathway, affected TDP-43 and the global SG marker Human Antigen R (HuR), others were more selectively effective against TDP-43 accumulation. Notably, inhibitors of cyclin-dependent kinases (CDKs) and glycogen synthase kinase 3 (GSK3) were identified, providing novel perspectives for targeting TDP-43 proteinopathy in drug development. Furthermore, Parker et al. utilized paraquat as a chemical stressor and observed that after overnight treatment, endogenous TDP-43 stably accumulates in SGs within HeLa cells [84]. This crucial finding underscores the propensity for protein aggregation in neurons affected by TDP-43 proteinopathy and highlights the diagnostic potential of detecting endogenous TDP-43 protein. The early application of inhibitors for extracellular regulated protein kinases (ERK) and c-Jun N-terminal kinase (JNK) prevented the formation of TDP-43 positive SGs, though they could not reverse the aggregations under prolonged stress. These insights are invaluable for the early diagnosis and therapeutic intervention of the disease.

#### 2.1.3 Targeting phosphorylated TDP-43

Phosphorylation of TDP-43 is the most identified marker of TDP-43 proteinopathy, affecting its aggregation through diverse post-translational modifications and is known for its neurotoxic effects [85]. Kinases target several specific residues within the C-terminal domain of TDP-43 for phosphorylation. Notably, phosphorylation at the S409/S410 epitope has been implicated in promoting abnormal oligomerization and fibril formation *in vivo*, highlighting its critical role in the disease process [86].

Rojas et al. developed a screening model for phosphorylated TDP-43 pathology using SH-SY5Y cells, inducing endogenous TDP-43 phosphorylation through glutathione depletion facilitated by ethacrynic acid (EA) [87]. They screened 23 compounds based on the 6-mercaptopurine scaffold, derived initially from CDC7 inhibitors, known for their selectivity and brain permeability. These CDC7 inhibitors effectively reduced TDP-43 phosphorylation in both human cells and an *in vivo C. elegans* model expressing human TDP-43, with one compound notably decreasing the severity of clinical symptoms in ALS disease models. Currently, a range of kinase inhibitors are in preclinical and clinical phases, targeting the reduction of TDP-43 phosphorylation. These include inhibitors for Casein kinase 1 (CK1), Cell division cycle 7 (CDC7), Protein tyrosine kinase 2 (PTK2), Tau tubulin kinase 1 and 2 (TTBK1/TTBK2), MAP4K, MAP2K, JNKs, and Protein kinase RNA-activated (PKR), and others [88]. This focus on modulating TDP-43 phosphorylation marks a significant area of interest in ALS drug development [89].

#### 2.1.4 Targeting TDP-43 nucleocytoplasmic distribution

In ALS, disease progression is marked by increased reactive oxygen species (ROS). Liu et al. discovered that TDP-43 mislocalization in the cytoplasm initiates a positive feedback loop involving the activation of AMPK and increased ROS production. This finding underscores the potential of the ROS/AMPK pathway and pathogenic TDP-43 mislocalization as critical targets for treating TDP-43 proteinopathy [90]. Additionally, they found that activating the A2A adenosine receptor (A2AR) can suppress AMPK activation and correct TDP-43 mislocalization. Leveraging this mechanism, they established a ROS-induced TDP-43 mislocalization model to identify A2AR agonists, aiming to discover compounds that mitigate TDP-43 proteinopathy.

#### 2.1.5 Targeting TDP-43 clearance

Cellular protein quality control mechanisms ensure that newly synthesized proteins achieve correct folding. This critical folding or refolding process is facilitated in the endoplasmic reticulum (ER) by chaperones, such as heat shock proteins. Within this context, both the unfolded protein response (UPR) and heat-shock response (HSR) play pivotal roles in maintaining protein homeostasis [91]. Any proteins that misfold or aggregate are targeted for removal *via* protein degradation pathways, which are crucial for preserving cellular protein equilibrium. The two primary systems responsible for this degradation are the ubiquitin-proteasome system (UPS) and the autophagy-lysosomal pathway [92]. Byung-Hoon Lee and colleagues identified USP14, a proteasome-associated deubiquitinating enzyme, as a regulator that can impede the degradation of ubiquitin-protein conjugates *in vitro* and within cells. This observation suggests that inhibitors of USP14 could potentially augment proteasome activity [93]. To explore this possibility, they screened 63,052 compounds to find inhibitors that could suppress USP14, using proteasomes reconstituted with USP14 and employing Ub-AMC as the assay substrate. They discovered a promising inhibitor, IU1, which enhanced proteasome activity and notably improved the clearance of overexpressed TDP-43.

Lucia Cragnaz and her team developed a cellular model for screening compounds that could address TDP-43 aggregation using HEK293 cells engineered to express EGFP-TDPF4L-12XQ/N under tetracycline control [94]. This model features mutations in the RNA recognition motifs RRM1 and RRM2, impairing the protein's splicing activity, and a region rich in asparagine and glutamine residues, promoting TDP-43 self-aggregation. They discovered that compounds such as nortriptyline and thioridazine enhance clearance through mechanisms that rely on an active proteasome pathway yet are independent of the later stages of autophagy. Additionally, Barmada et al. crafted and validated a live cell autophagic flux assay to identify strong autophagy inducers. This assay used primary murine neurons expressing WT and mutant TDP-43-Dendra2 (A315T) [95]. Leveraging an *in-silico* library of over 1 million compounds, they successfully identified three compounds that significantly boost the clearance of both WT and mutant TDP-43-Dendra2 in primary neurons by stimulating autophagy.

#### 2.1.6 Summary

Due to their ease of culture, rapid growth rates, and straightforward genetic modifiability, numerous specialized cell lines have been developed to investigate TDP-43 proteinopathy. These cell lines are designed to target various pathological mechanisms, such as directly lowering TDP-43 levels, disrupting TDP-43 protein-protein interactions, recruiting TDP-43 into SGs, forming insoluble TDP-43 aggregates, modulating TDP-43 phosphorylation, and enhancing the clearance of pathological TDP-43. Utilizing a range of analytical instruments, these cell lines enable the screening of diverse compound libraries *via* high-content and/or high-throughput drug screening systems, offering a pathway to discover novel potential therapeutic agents. However, based on isolated single cells, these cell models have inherent limitations in their completeness and representativeness. Given the complex, systemic nature of ALS, there is a critical need for more comprehensive drug screening and validation models that can better mimic the disease's multifaceted pathology.

### 2.2 *In vivo* drug screening models

Model organisms are frequently employed in biological research due to their physiological attributes, genomic configurations, and life cycles, which are representative of a broad spectrum of biological phenomena and fundamental mechanisms. In the context of TDP-43 proteinopathy, scientists have developed several *in vivo* drug screening models utilizing a diverse array of model organisms. These include *Caenorhabditis elegans* (*C. elegans*), Drosophila, *Danio rerio* (zebrafish) and yeast, each chosen for their unique ability to elucidate different aspects of the disease and facilitate the evaluation of potential therapeutic compounds.

#### 2.2.1 C. elegans

*C. elegans* is extensively utilized in life sciences and medical research due to its conserved regulatory mechanisms for various biological processes, closely mirroring those in humans. This makes it an excellent model for studying ALS, particularly in the context of TDP-43 proteinopathy [96]. The advantages of using *C. elegans* include its ease of cultivation, rapid growth and reproduction rates, abundant genetic resources, and the availability of ideal mutant strains, all of which significantly contribute to its effectiveness in ALS research. Furthermore, *C. elegans* was the first multicellular organism to have its entire genome sequenced, and a comprehensive genome-wide RNAi feeding library is available for use. This facilitates not only conventional molecular compound drug screening but also enables high-throughput RNAi screening, broadening the scope of potential therapeutic discoveries [97].

To explore TDP-43 proteinopathy in ALS, Vaccaro et al. conducted representative transgenic *C. elegans* models that express either the human full-length WT TDP-43 or mutant TDP-43 (mTDP-43) specifically in the worm’s GABAergic MNs [98]. These models develop adult-onset, age-dependent progressive paralysis and motor neuron degeneration, mirroring critical aspects of ALS. Notably, the mTDP-43 strains, unlike the WT TDP-43 strains, accumulate insoluble proteins within the whole organism, closely replicating human pathological conditions and validating the model's fidelity. Leveraging these models, Vaccaro et al. refined a liquid culture technique that enhances the early detection of paralysis and other ALS-associated pathological features, laying the groundwork for effective drug screening. This methodology identified methylene blue as a robust inhibitor of TDP-43-induced toxicity [99, 100]. Bose et al. utilized the same liquid culture technique for high-throughput screening of 3,768 compounds using TDP-43 A315T transgenic *C. elegans*, identifying a promising new small molecule, TRVA242 [101]. In addition to transgenic worm models expressing WT, A315T or G348C mutant TDP-43 respcctively, Liachko et al. reported another transgenic worm model expressing M337V mutant TDP-43. This model was used in conjunction with a comprehensive RNA interference (RNAi) library designed to target kinases involved in TDP-43 phosphorylation [102]. Through RNAi screening, they identified CDC7 among eleven other kinases that, when inhibited, ameliorated movement deficits in the models. Furthermore, the small molecule inhibitor PHA767491 significantly decreased TDP-43 phosphorylation and effectively mitigated TDP-43-related neurodegeneration.

While *C. elegans* presents distinct advantages as an ALS model, particularly for studying TDP-43 proteinopathy, it is critical to recognize that this nematode, like all animal models, cannot encapsulate the full spectrum of the human condition. As an invertebrate, it lacks certain vertebrate-specific characteristics, including a blood-brain barrier (BBB), which poses challenges for drug efficacy studies relative to mammalian models. Furthermore, while useful, the genetic similarity between *C. elegans* and humans is not comprehensive, restricting the extrapolation of results to human conditions. These limitations underscore the necessity for employing a range of complementary models to gain a complete understanding of ALS pathogenesis and to facilitate the discovery of effective treatments.

#### 2.2.2 Yeast

Yeast serves as a potent model organism for drug screening, benefiting from its robust growth on basic medium and significant genomic homology with humans. This similarity ensures that many core cellular pathways are preserved, enhancing the relevance of genetic interactions observed in yeast to human diseases [103]. The well-documented and easily modifiable yeast genome offers a rich array of genetic tools. These resources are invaluable for uncovering pathways and genes impacted by the pathological accumulation of disease-associated proteins, thereby facilitating targeted therapeutics development [104].

To explore modifiers of TDP-43 toxicity, Armakola et al. developed a yeast system overexpressing WT TDP-43 using an integrated galactose-inducible TDP-43 construct (pAG303Gal-TDP-43). Utilizing the Yeast FLEXGene ORF library, which includes approximately 5500 yeast open reading frames, they set out to identify genes that either suppress or enhance TDP-43 toxicity [105]. This involved overexpressing each gene from the library in yeast cells carrying the TDP-43 construct and then inducing gene expression by plating the cells on galactose media. This methodical screening process identified 40 genetic modifiers, encompassing both enhancers and suppressors of toxicity. Notably, PBP1, a yeast homolog of human ataxin 2, was found to increase the risk for ALS significantly and was pinpointed as a critical target for drug development efforts. Additionally, Park et al. demonstrated that the yeast gene TIP41 deletion also curtailed TDP-43 toxicity [106]. The beneficial effects of these genes were linked to the activation of autophagy, highlighting potential therapeutic pathways.

To further elucidate, Peggion et al. advanced our understanding of genetic modifiers of TDP-43 toxicity by developing a sophisticated yeast model for TDP-43 proteinopathy [107]. In their approach, they activated TDP-43 expression through a glucose/galactose switch and engineered yeast strains with varying copy numbers of WT TDP-43 (single (TDP 1C), double (TDP 2C), or triple (TDP 3C) copies). Their findings revealed that TDP-43 expression altered cell morphology and that yeast strains with more than two copies exhibited a marked increase in insoluble TDP-43 protein levels, intensifying cytotoxic effects. Additionally, the ALS-associated missense mutations Q331K and M337V further heightened TDP-43 cytotoxicity. In this model system, the nucleolar protein nucleolin emerged as a significant inhibitor of TDP-43 toxicity, effectively promoting the nuclear retention of TDP-43 and mitigating the formation of deleterious cytosolic TDP-43 inclusions. Exploring oxidative stress mitigation provides a novel avenue for developing therapeutics targeting TDP-43 proteinopathy. In yeast models, TDP-43 overexpression has been shown to elevate oxidative stress levels markedly. Bharathi et al. crafted an oxidative stress model utilizing the ade1 or ade2 mutant yeast's red-white colour reporter system [108]. Employing this red-white reporter yeast assay along with CellROX staining, they illustrated that overexpression of Sis1 could significantly reduce the oxidative stress and cytotoxicity induced by TDP-43. In addition to these methods, yeast deletion screens, also known as synthetic lethal screens, serve as a valuable tool for detecting genetic interactions that reveal connections between ostensibly unrelated genes or pathways. Through these screens, Armakola and colleagues identified 14 genetic modifiers that influence TDP-43 toxicity, which include eight suppressors that alleviate toxicity and six enhancers that increase it. Importantly, they discovered that the most potent suppressive gene deletion involved DBR1, which encodes the RNA lariat debranching enzyme, a protein that is conserved from yeast to humans. This finding underscores the potential of DBR1 as a key target in reducing TDP-43 toxicity [105].

Developing yeast models has significantly advanced drug development and gene therapy efforts for TDP-43 proteinopathy, though these models are not without their limitations. For instance, in screening for enhancers or suppressors in TDP-43-overexpressed yeast models, interfering with genes in the promoter region can skew the results. Furthermore, yeast models cannot replicate ALS-associated phenotypes such as motor function impairment and MN loss, which is a significant limitation compared to other model organisms. Despite these challenges, the extensive suite of genetic tools available in yeast models proves invaluable for identifying genes regulated by TDP-43. This capability furnishes a plethora of potential targets for drug development, underscoring the utility of yeast in the ongoing search for treatments for TDP-43 proteinopathy.

#### 2.2.3 Drosophila

Drosophila is a classic model organism with several advantages, including ease of maintenance, rapid reproductive cycles, and large progeny numbers. Approximately 75% of human disease genes have homologs in Drosophila [109, 110]. In the realm of NDD, Drosophila exhibits numerous analogous phenotypes, including late-onset manifestations, progressive degeneration, and significant neural system toxicity. Therefore, integrating the Drosophila model into the construction of ALS disease models targeting TDP-43 proteinopathy is highly appropriate [111].

The overexpression of TDP-43 (either WT TDP-43 or mTDP-43) in MNs induces nearly complete pupal lethality, with only a handful of pharate adults emerging, incapable of extending their wings [112]. Building on this critical observation, Joardar et al. implemented a drug screening approach using the GAL4-UAS system to drive the expression of human TDP-43, including both the WT and disease-associated mutants (D169G, G298S, A315T, and N345K) in MNs [112]. They utilized the pupal lethality phenotype as a primary screening parameter to identify drugs that could potentially reverse this lethality and allow for the emergence of viable adults with extended wings. Ultimately, several antidiabetic medications were discovered, notably pioglitazone, known for their neuroprotective properties. Beyond targeting merely lethal or nonviable pupal phenotypes, Piccolo et al. devised an innovative Drosophila-based drug screening platform. This involved a Drosophila strain engineered with a TDP-43 G384C mutation, effectively mirroring various facets of TDP-43 pathology [113]. The evaluation focused on enhancements in eclosion rates and assessments of locomotive abilities at both early and late developmental stages. Through this comprehensive approach, Fingolimod stood out as a potent agent for mitigating TDP-43 toxicity.

Beyond conventional pharmacological approaches, gene therapy has been explored using Drosophila models. In these models, TDP-43 expression is intricately controlled by an autoregulatory feedback mechanism within its mRNA 3’UTR, known as TDPBR, offering a viable therapeutic target. Pons et al. created a TDP-43_TDPBR Drosophila model that replicates crucial aspects of this TDP-43 feedback loop [114]. This innovative model employs the expression of WT TDP-43 complementary DNA regulated by the TDPBR region, which aids in pinpointing genetic modulators that impact TDP-43 production levels. Researchers have successfully identified such modulators using this model. Investigating these genes’ human analogues may lead to new therapeutic avenues and potential drug discoveries.

While the Drosophila model offers distinctive benefits, its simplicity in biological structure, akin to that of the nematode model, limits its capacity to accurately mimic and investigate the intricate neuropathology and mechanisms prevalent in human diseases. Moreover, the substantial variances in DNA sequences and protein architectures between Drosophila and humans curtail its widespread use in certain research domains. These constraints underscore the imperative for auxiliary model systems and the adoption of diversified methodologies to grasp the complexities associated with human diseases thoroughly.

#### 2.2.4 Danio rerio

Zebrafish have emerged as a compelling model organism for various scientific disciplines based on several advantageous traits. Their small size, low maintenance cost, and capacity for large-scale breeding make them a viable alternative to traditional model organisms [115]. As a non-mammalian vertebrate, zebrafish share a closer genetic and physiological similarity to humans than invertebrate models such as *C. elegans* and Drosophila, making them a crucial resource for studying vertebrate developmental biology and increasingly favoured for applications in other areas. One of the unique benefits of using zebrafish in NDD research is their possession of a BBB that resembles that of higher vertebrates, providing a significant advantage over models like Drosophila, yeast, and *C. elegans* [116]. These attributes have positioned zebrafish at the forefront of ALS research, facilitating the development of various TDP-43 proteinopathy models to advance drug discovery.

Vaccaro et al. and Bose et al. have developed a transgenic zebrafish model for GOF screening, analogous to the *C. elegans* model, to investigate TDP-43 proteinopathy [99, 101]. In this approach, human TDP-43 G348C mRNA is pulse-injected into zebrafish embryos at the 1-2 cell stage. By 48 hours post-fertilization, the resulting larvae exhibit locomotor deficits, specifically a reduced ability to perform a touch-evoked escape response. This model has been effectively utilized in ALS drug discovery efforts, culminating in identifying a promising new compound, TRVA242, which exhibits potential neuroprotective effects [101].

Although the zebrafish model presents distinctive benefits, it is important to recognize its limitations due to the lack of complexity in human organs and systems, which hampers its ability to decode the intricacies of systemic diseases completely. Moreover, the aquatic environment necessary for zebrafish development can restrict certain types of drug testing, especially with poorly soluble compounds in water, potentially compromising their assessment in this model. Consequently, while zebrafish offers valuable preliminary insights, it is imperative to integrate it with other model systems and methodologies to achieve a thorough understanding of disease mechanisms and to enhance drug development strategies.

#### 2.2.5 Summary

In conclusion, each *in vivo* drug screening model possesses unique strengths and weaknesses. Although these models are instrumental in uncovering disease mechanisms and identifying potential therapeutic compounds, they fail to capture the complexity of human pathologies and pharmacological responses fully. Consequently, it is essential to utilize more sophisticated drug validation models that rigorously evaluate the protective impacts of these compounds against TDP-43 proteinopathy. Such models are crucial for a more detailed assessment of drug effectiveness, safety, and potential adverse effects, which are vital steps before transitioning to human clinical trials.

### 2.3 ALS patient-derived iPSCs-MNs

Recent developments in stem cell technology, especially the creation of induced pluripotent stem cells (iPSCs), have significantly broadened the prospects for investigating ALS and developing new therapeutic strategies. ALS predominantly impacts MNs, which are crucial for relaying signals from the brain to the muscles to facilitate voluntary movements. iPSCs generated from individuals with ALS can be differentiated into MNs, thus establishing an *in vitro* model that authentically replicates the disease’s neuropathology. This neuronal model offers enhanced capabilities to investigate the mechanisms and progression of pathological changes arising from disrupted neuronal function, surpassing traditional cell models. Furthermore, compared to model organisms, iPSC-derived MNs (iPSCs-MNs) have the distinct advantage of being patient-specific. They preserve the exact genetic constitution of the individual, including any mutations implicated in the disease, providing a more precise platform for understanding ALS [117, 118]. Given these unique properties, iPSCs-MNs serve as a potent alternative to conventional *in vivo* and *in vitro* models and excel as a drug validation model. This makes them exceptionally valuable for assessing the efficacy and safety of new therapeutic compounds before they proceed to clinical trials.

#### 2.3.1 ALS TDP-43 MNs showed ALS-related phenotypes.

Through gene editing, scientists generated iSPCs from fibroblasts and peripheral blood [119] of ALS patients with various mutations in TDP-43, including M337V [58, 120, 121] Q343R [58, 121], A382T [119, 122], N352S [65], I383V [123, 124] and G298S [58, 65, 123, 124]. Most of these differentiated mutant MNs exhibited some common TDP-43 proteinopathy characteristics, including TDP-43 mislocalization into the cytoplasm, cytoplasmic TDP-43 aggregation and increased levels of phosphorylated TDP-43 with or without stress [121, 123, 124]. Matthew et al. established a cellular model for sporadic ALS using patient-derived iPSCs, which showed TDP-43 aggregation recapitulating pathology in postmortem tissue from the same patient [125]. Simultaneously, utilizing lymphoblastoid cell lines and human lymphocytes from sporadic ALS patients, Koki et al. [121] and Gabriel et al. [123] developed numerous *in vitro* cellular models that exhibit phenotypes associated with ALS. These include shortened neurites, neurite swelling, SGs formation, mitochondrial dysfunction, oxidative stress, lactate dehydrogenase (LDH) leakage, and MN death. These models provide a functional representation of the disease at the cellular level. iPSC-derived MNs (iPSCs-MNs) further extend these capabilities by accurately replicating ALS's abnormal cellular and molecular features *in vitro*, offering a robust platform for studying the disease's underlying mechanisms and testing potential therapeutic interventions.

#### 2.3.2 Drug screening based on ALS patient-derived iPSCs-MNs

Recognizing that arsenite can induce oxidative stress and elevate the levels of insoluble TDP-43 in MNs, Egawa et al. crafted an assay to assess the mortality of ALS iPSC-derived MNs (iPSCs-MNs) following arsenite exposure [126]. They determined the survival rate of these neurons (measured as the proportion of HB9: GFP-positive cells) to identify drugs that mitigate arsenite-induced neuronal death. Their research revealed that anacardic acid significantly reduced TDP-43 mRNA expression in ALS iPSC-derived MNs by 147-fold relative to untreated cells and decreased the levels of insoluble TDP-43. Additionally, the formation of SGs contributed to the persistent cytoplasmic accumulation of TDP-43 in puncta within the MNs.

To explore whether compounds that modulate the formation of SGs could diminish persistent cytoplasmic TDP-43 puncta in puromycin-stressed iPSCs-MNs, Fang et al. differentiated both control and ALS-specific iPSC-MN lines bearing TDP-43 mutations N352S and G298S. These ALS-associated iPSC-MNs demonstrated distinct phenotypes indicative of the disease [81]. By quantitatively measuring the ratio of cytoplasmic TDP-43 puncta area to nuclear area, Fang et al. identified that compounds with planar structures effectively reduce cytoplasmic TDP-43 puncta, thereby mitigating the ALS-associated phenotypes. Compared to models induced by arsenite and puromycin, iPSC-MNs derived from sporadic ALS (sALS) patients exhibit intranuclear TDP-43 aggregation even without external stressors. Burkhardt and colleagues [127] employed iPSC-MNs from three sALS patients for a high-content chemical screening, ultimately identifying four drug classes, including Digoxin, that significantly reduce the percentage of cells with TDP-43 aggregates in a dose-dependent manner in sALS patient iPSC-MNs. This breakthrough highlights the potential of these compounds to counteract one of the hallmark features of ALS pathology.

#### 2.3.3 Drug validation based on ALS patient-derived iPSCs-MNs

The iPSCs-MNs model, as a complex system, can be used not only for drug screening but also as a major model for drug validation. Both TDP-43 proteinopathy and ALS-related phenotypes displayed in the iPSCs-MNs model make it widely used for ALS drug development. Barmada et al. screened and found three compounds which can reduce the expression of TDP-43 *via* the autophagy-lysosome pathway [95]. Then, ALS TDP-43 M337V MNs and astrocytes harbouring TDP-43 M337V are used to confirm that autophagy inducers can mitigate TDP-43 aggregation and insoluble proteins. Fujimori et al. developed a multi-phenotype screening system with a high-content imaging system. The multi-phenotype screening system includes neurite lengths, mitochondria dysfunction, the number of formed SGs, FUS aggregation, and LDH leakage. MNs from FUS ALS iPSCs, TDP-43 ALS iPSCs and non-SOD1 FALS were used to ultimately identify a compound named ropinirole (ROPI) as a potential candidate for the ALS treatment [121]. Hung et al.[124] and Linares et al.[123] identified PIKFYVE kinase and SYF2 as therapeutic targets through a phenotypic screening to enhance survival in C9ORF72 ALS/FTD-induced MNs (iMNs). They demonstrated that inhibition of these targets could mitigate ALS pathology and prolong survival in animal models and iPSCs-MNs models from various genetic backgrounds carrying mutant *C9orf72*, *TARDBP*, and *FUS*, and sporadic cases of ALS.

#### 2.3.4 Summary

Recent advancements in gene editing technologies like CRISPR-Cas9, alongside the evolution of intricate 3D culture systems, are revolutionizing disease modelling and drug discovery. Numerous iPSC-MNs models have been developed for ALS drug research. Nevertheless, clonal variation presents significant challenges, with noticeable differences even among iPSC lines derived from the same patient [128]. These variations, coupled with the intrinsic limitations of gene editing techniques and potential off-target effects, hinder the efficacy of model development and its practical applications. Further complicating the landscape, the diversity in differentiation protocols across different laboratories leads to considerable discrepancies in the disease phenotypes and onset timing of the differentiated MNs. This variability complicates the standardization of the model for broader use [129]. Other concerns include the ethical and moral implications associated with using human genetic material and the logistical challenges of MN differentiation, namely the extended timeframes, high costs, and stringent laboratory requirements needed to maintain culture conditions. Due to these issues, iPSC-MN models are often relegated to later stages of drug testing, primarily for validating the efficacy of pre-existing compounds rather than for pioneering new high-content or high-throughput screening methods. Despite these obstacles, the integration of various models and the strategic combination of different methodologies continue to drive forward ALS research and drug development.

## 3. Drug validation models for ALS

Creating an exemplary disease model is a formidable task. It requires a model that can accurately replicate the pathological changes observed in human diseases, ensuring that these disease phenotypes are stable and reproducible. Additionally, the genetic information and tools available for the organism used as the model must be comprehensive to support various genetic interventions. Moreover, the costs associated with breeding, maintenance, research, and development are also critical considerations. Therefore, choosing a suitable drug validation model is fundamental to the success of ALS drug development efforts. This review also aims to provide a comprehensive overview of the numerous TDP-43 proteinopathy drug validation models currently available.

### 3.1 Common model organisms

Several widely utilized model organisms provide key advantages over cellular and larger animal models, particularly in demonstrating ALS-associated phenotypes and their ease of breeding. These models are integral not only for initial drug screening processes but also for preliminary assessments of drug efficacy. For example, Alexandra et al. developed multiple transgenic nematode models that involved overexpressing mTDP-43 proteins specifically within the D-type MNs of nematodes [98]. Marte et al. found the cytotoxicity of the mTDP-43 overexpression model in yeast [130]. Overexpression of TDP-43 M322K mutation in yeast can significantly increase protein aggregation and cytotoxicity, and the prominent pathological characteristics make it an ideal model for drug validation. Zebrafish possess two homologous genes, *tardbp* and *tardbp-like* (*tardbpl*), comparable to the human *TARDBP* gene. Consequently, an ALS disease model can be constructed by overexpressing these orthologous genes. Asakawa et al. developed an optogenetic TDP-43 variant (opTDP-43, *tardbp*) zebrafish model, demonstrating that that long-term stress stimulation accelerates disease progression [131]. Furthermore, Bose et al. and Kabashi et al. have developed a transgenic zebrafish model using loss-of-function screening techniques facilitated by CRISPR/Cas9 gene-editing technology [101, 132]. They engineered models lacking the *tardbp* and *tardbpl* genes. By breeding two gene-edited adult zebrafish, they produced a cohort of embryos homozygous for deletions in both *tardbp* and *tardbpl* (*tardbp*^-/-^*tardbpl*^-/-^). These double homozygous mutants proved to be lethal, exhibiting symptoms such as muscle degeneration and a reduction in the length of spinal motor neuron axons, thereby contributing another model for ALS research. Table 1 offers a detailed overview of the pathological and behavioral traits observed in commonly used model organisms that are pertinent for verifying drug efficacy.

**Table 1:** Disease models and major phenotypes of ALS in common model organisms.

Model organism	Type	Major Disease Phenotypes	Reference
*C. elegans*	Wild type TDP-43	1)Progressive paralysis 2)Adult-onset, age-dependent loss of motility 3)Degeneration of MNs 4)Oxidative stress 5)Insoluble proteins aggregation^1^	[98, 99]
	TDP-43 A315T mutation		
	TDP-43 G348C mutations		
	TDP-43 M337V mutations		[102]

Yeast^2^	Wild type TDP-43	1)Decreased growth rate. 2)Cellular toxicity 3)Insoluble protein aggregation 4)Shape defect of the yeast strains 5)Oxidative stress	[105]
	Different copies of Wild type TDP-43		[107]
	TDP-43 Q331K mutations		
	TDP-43 M337V mutations		
	TDP-43 M322K mutations		[130]

*Drosophila*	Wild type TDP-43	1)Pupal lethality^3^ 2)Decreased eclosion rate. 3)Motor capacity is reduced in early and late developmental stages. 4)Decreased life span. 5)Neuromuscular junction abnormalities 6)Insoluble protein aggregation	[112]
	TDP-43 D169G mutations		
	TDP-43 G298S mutations		
	TDP-43 A315T mutations		
	TDP-43 N345K mutations		
	TDP-43 G348C mutations		[113]
	TDP-43_TDPBR		[114]

*Danio rerio* ^4^	Wild type op*tdrdbp*	1)Muscle degeneration 2)Vascular defects 3)Aberrant spinal motor neuron outgrowth, and premature death 4)Neuromuscular junction abnormalities	[131]
	*tardbp* ^-/-^ *tardbpl* ^-/-^		[132]
	TDP-43 G348C mutations		[101]

Mutant TDP-43 proteins are highly insoluble, while wild-type proteins remain soluble.

2 More than two copy numbers of TDP-43 to show the disease phenotype.

3 In some disease models, 100% pupal lethality and few pharate adults eclose but cannot extend their wings.

4 The disease phenotypes of single homologous gene knockout are not obvious.

### 3.2 Rodent

Rodents, as mammals, share a close genetic and physiological relationship with humans, which fundamentally underpins their utility as model organisms in medical research. The mouse has become the predominant model for ALS, particularly for drug validation. Numerous TDP-43 mouse models, including those that overexpress either WT or mutant forms of TDP-43, have been established to elucidate its role in ALS pathology [51, 133, 134]. In addition, models featuring TDP-43 loss-of-function through conditional deletion in MNs have also been developed [135]. These TDP-43 mouse models effectively replicate several critical aspects of ALS observed in humans, such as weight loss, muscle atrophy, loss of MNs, and the accumulation of ubiquitinated and phosphorylated TDP-43 inclusions within the cytoplasm [51]. Giorgio et al. provide a detailed overview of these models and their significance, which is invaluable for researchers in the field [136]. Here, we aim to outline several key ALS TDP-43 mouse models and review some of the drug tests performed using these models. Further, we intend to serve as a resource for forthcoming drug development and exploring new therapeutic strategies for ALS.

The Prnp-*TARDBP* A315T mouse model, established by Stallings et al., is frequently used to study mislocalized TDP-43 pathology [137]. This model harbours a modified full-length human TDP-43 with the A315T mutation, exhibiting an average lifespan of 3.5 months in males, though females typically survive longer [138]. The TDP-43 A315T mouse has been widely utilized in numerous drug screening and validation experiments. Kumar et al. discovered that IMS-088, a novel analogue of withaferin-A, effectively reduces TDP-43 proteinopathy in both the brain and spinal cord of this transgenic mouse model by promoting autophagy and improving translational deficiencies [139]. Additionally, Martínez-González et al. used the TDP-43 A315T model to demonstrate that Tideglusib could lower phosphorylated TDP-43 levels and restore its nuclear localization, findings that recently propelled this compound into clinical trials [140].

An alternative TDP-43 disease model utilizing the well-characterized *TARDBP* M337V mutations has also been explored, demonstrating progressive mobility impairment and MN degeneration [137]. Successive iterations of TDP-43 M337V mouse models, varying in genetic backgrounds and construction methodologies, have similarly replicated ALS phenotypes, serving as valuable tools in pathogenesis studies and therapeutic interventions [141-143]. Huang et al. advanced this line of research by introducing the TDP-43 M337V virus into the striatum of mice, both unilaterally and bilaterally, to establish a mouse model of ALS characterized by TDP-43 proteinopathy. This model was used to investigate the effects of Tetramethylpyrazine nitrone (TBN). Their findings indicated that TBN enhanced motor function and extended survival by activating Akt/mTOR/GSK-3β and AMPK/PGC-1α/Nrf2 signalling pathways. Significant challenges such as rapid disease progression and premature mortality are typical in TDP-43 mouse models that harbour genetic mutations under the control of the murine prion promoter [143]. Another significant model is the *TARDBP* Q331K transgenic mouse, initially developed by Arnold et al. in 2013. This model, which also carries a mutation identified in ALS patients, was later backcrossed onto a C57BL/6N genetic background by Jackson Laboratories, enhancing its utility for research purposes [145].

Despite the development of numerous knock-in mouse models that overexpress mutant TDP-43, not all exhibit ALS-like phenotypes, with some only displaying neurodegenerative features in the disease's late stages. Recently, advancements have been made with several mouse models that express WT TDP-43, demonstrating dose-dependent phenotypes correlated with TDP-43 pathology. Wils et al. developed a transgenic mouse line that expresses human WT TDP-43, revealing that its overexpression leads to motor neuron degeneration in both the spinal cord and cortex [51]. Similarly, Xu et al. created another transgenic mouse that expresses the full-length human WT TDP-43. This model showed that TDP-43 overexpression results in protein truncation, increased ubiquitin levels, and cytoplasmic accumulation of phosphorylated TDP-43 [146]. These findings underscore that the level of TDP-43 expression is critically linked to the disease's onset and severity. In 2015, Walker et al. introduced a novel ALS mouse model named rNLS8 expressing WT TDP-43 [147]. This model leverages the neurofilament heavy chain (NEFH) promoter to regulate the expression of human WT TDP-43, engineered with a defective nuclear localization signal (ΔNLS). Expression of this TDP-43 variant is controllable *via* Doxycycline (DOX); cessation of DOX treatment induces ALS-like phenotypes, including the formation of TDP-43 inclusions, rapid motor neuron degeneration, and deteriorating motor functions. The rNLS8 model's inducible expression of TDP-43 pathology and its consistent phenotypes render it an excellent tool for exploring treatments that aim to lessen cytoplasmic TDP-43 aggregation, address TDP-43-related disturbances, and reinstate its normal nuclear functions. Recent studies utilizing the rNLS8 model have demonstrated that several novel monoclonal antibodies targeting the glycine-rich domain of TDP-43 reduce its liquid-liquid phase separation and aggregation effectively, thereby showing promise in slowing disease progression [148, 149]. However, it is important to note that Riluzole, a standard clinical drug for ALS, failed to exhibit any protective effects in this model, underscoring the inherent complexity and the significant challenges in developing effective treatments for ALS [150].

Rodent models, such as mice, are indispensable in ALS research for their genetic similarities to humans and their capacity to replicate ALS disease phenotypes, including TDP-43 pathology. By incorporating ALS-related mutations into these models, researchers can more accurately study the disease’s pathogenesis and potential treatments at a manageable cost. However, no disease model perfectly mirrors human conditions. For instance, the *TARDBP* A315T mouse model often exhibits gut immobility due to increased TDP-43 expression in the myenteric plexus and subsequent neuronal degeneration [151, 152], complicating its utility for drug screening. In our research, certain male rNLS8 mice have developed conditions such as uroschesis and overgrown teeth, which interfere with feeding and complicate the assessment of late-stage disease manifestations. Moreover, the variability in animal experiments and differing care conditions across laboratories can introduce significant variability, even within the same mouse strain. Despite these challenges, the mouse model remains critical in ALS research. Its thoughtful application can significantly enhance our understanding of ALS and foster the advancement of treatments, underscoring its vital role in the continuum of complex disease research.

### 3.3 Large mammalian models

Large animal experiment is an experimental method that is widely used in scientific research and medicine. It uses large animals (such as pigs, monkeys and others) as models to study various diseases and test drugs. The physiological structure and physiological function of large animals are more similar to humans, so they have higher reliability and accuracy in some disease simulations and drug testing [153]. Moreover, considering the physiological and metabolic reactions in pharmacokinetics, the body size and metabolic system of animals are closer to humans, so the drug development data obtained from them are more reliable [154]. At the same time, compared with human clinical experiments, extensive animal experiments can be carried out in a controlled environment, including diet, exercise, and environmental factors. Thus, the influence of external factors on the experimental results can be reduced. Therefore, large animal models have also been developed for the drug-effect study of ALS.

Mammalian disease models of TDP-43, especially those utilizing monkeys and pigs, are superior to smaller animal models because they mirror human anatomy and genetics more closely. A critical pathological feature of ALS is the cytoplasmic accumulation of TDP-43 protein. However, in many transgenic TDP-43 rodent models, TDP-43 predominantly localizes in the nucleus within the brain rather than the cytoplasm. In contrast, Yin et al. observed that the introduction of the same adenovirus AVV-TDP-43 M337V into the right substantia nigra of both rhesus monkeys and mice elicited distinct patterns of subcellular TDP-43 distribution, illustrating the differential cellular responses in larger versus smaller mammals [155]. In rhesus monkeys, cytoplasmic aggregation of TDP-43 was notably evident, a phenomenon potentially attributable to the primate-specific caspase-4. Unlike its mouse counterpart, caspase-11, caspase-4 can cleave the NLS-containing N-terminal domain of TDP-43, leading to its fragmentation and mislocalization to the cytosol. This primate-specific cleavage mechanism by caspase-4 underscores its potential as a target for drug development, offering insights into the pathogenesis of TDP-43 in primate brains. In parallel, Wang et al. utilized mutant TDP-43 M337V to transfect primary pig fetal fibroblasts and subsequently created a transgenic pig model through somatic cell nuclear transfer technology [156]. In this model, mutant TDP-43 protein was predominantly localized in the cytoplasm of neuronal cells. This mislocalization was associated with severe clinical phenotypes, including progressive muscular weakness, movement impairments in limbs, and a reduced lifespan, underscoring the model's utility in mimicking human disease and exploring therapeutic avenues. These observations from rhesus monkeys and transgenic TDP-43 minipigs provide critical data on TDP-43 pathology. They validate the importance of these larger mammalian models in ALS research and in identifying potential therapeutic targets.

Undoubtedly, conducting experiments using large animals involves significant costs and time commitments, including expenses for animal maintenance, experimental equipment, and personnel, all of which contribute to the heightened overall cost and extended duration of research and drug development projects. Moreover, ethical issues associated with extensive animal experiments are notably more complex than those involving humans, primarily because animals cannot give informed consent or voluntarily participate in studies. Despite these challenges, extensive animal experiments are indispensable tools in scientific research, playing a critical role in the medical field. They are crucial for gaining a more comprehensive understanding of the potential pathogenic mechanisms and pathological manifestations of ALS, thereby aiding in developing effective treatments for the disease.

## 4. Challenges for developing effective drugs against TDP-43 proteinopathy

NDD represent a spectrum of disorders marked by neuronal dysfunction, which ultimately leads to neuronal death and localized tissue degeneration [157]. Currently, managing NDD, including Alzheimer's disease and Parkinson's Disease, poses a considerable challenge, as existing treatments only manage to delay the onset of symptoms without arresting the progression of the disease [158, 159]. Similar challenges persist in the treatment of ALS. Despite the FDA approving three drugs to manage ALS, their clinical effectiveness remains substantially limited [160, 161]. This issue is intricately linked to the disease's complex pathogenesis. Scientists have proposed numerous potential pathogenic mechanisms for ALS, but a complete understanding of its underlying causes remains elusive, complicating the development of effective treatments.

Since 2006, the discovery of TDP-43 positive inclusions in ALS patients has underscored the protein's ubiquity and implicated it as a central element in the disease's pathogenesis. This revelation has intensified scientific focus on the crucial role of TDP-43 proteinopathy in ALS, leading to the development of numerous disease models associated with TDP-43 for drug screening and therapeutic development. Significant advancements have been achieved in identifying a wide array of drugs and small molecules to target TDP-43 proteinopathy, a body of work extensively reviewed by Buratti [162]. While some drugs targeting TDP-43 proteinopathy have advanced to clinical trials, a definitive cure for the condition has not yet been found. A critical analysis of the various models used for drug screening reveals several underlying issues. First, TDP-43 plays multiple roles in mRNA processing across both the nucleus and cytoplasm [163], which means that its dysfunction can contribute to neurodegeneration through both loss of function and toxic gain of function. Consequently, many disease models used for drug screening do not specifically isolate TDP-43, leading to the selection of compounds that might disrupt its normal function. While these compounds may reduce TDP-43 expression levels or their aggregation, they can be detrimental to normal cellular functions and, by extension, harmful to human health.

Secondly, assessing the translational potential and reliability of preclinical findings for numerous compounds remains a formidable challenge. Presently, there are no comprehensive guidelines to evaluate the safety and efficacy of treatments for TDP-43 proteinopathy. Such guidelines should cover a broad range of aspects, including the identification of active components, the establishment of dose-response and time-dependent curves, the evaluation of side effects, and the implementation of efficacy assessment models. For example, treatments derived from complex mixtures such as maple syrup require precise identification of the specific active ingredients that confer protective effects. In cases involving certain compounds like rapamycin, torin1, and other mTOR inhibitors, evidence supports their capacity to diminish TDP-43 aggregation and the presence of insoluble phosphorylated TDP-43 [164]. Nonetheless, the potential side effects of mTOR inhibition, particularly at therapeutic concentrations and during prolonged use, must be carefully considered. Although these inhibitors have shown promise in various disease models, they have not gained clinical approval due to ongoing safety concerns.

Thirdly, there is a notable deficiency in the drug screening and validation models. Over 50 missense mutations in the *TARDBP* gene have been identified [165-167], yet most drug screening models focus on just a subset of these mutations, which does not adequately capture the varied disease manifestations of TDP-43 proteinopathy. This limitation undermines the reliability of the compounds identified through these models. Moreover, ALS patients display a spectrum of symptoms related to TDP-43 proteinopathies, such as nucleocytoplasmic imbalances, dysregulation of post-translational modifications (including phosphorylation and cleavage), and disruptions in processes essential for the initiation and clearance of protein aggregation (like SGs, autophagy, and proteasomal activities) [168-170]. Existing *in vivo* and *in vitro* models do not comprehensively mimic all associated TDP-43 phenotypes, thereby constraining the dependability of drug development efforts. For instance, the most used ALS mouse models exhibit pathological discrepancies and significant individual variability that complicates drug development. Specific models, such as TDP-43 A315T mice, often suffer premature death due to intestinal blockages, while rNLS8 mice experience uroschesis issues, both of which severely limit the practical application of these models in experimental settings.

When identifying promising drug candidates, it is crucial to consider their ability to cross the BBB. The BBB effectively prevents nearly all large-molecule drugs and over 98% of small-molecule drugs from reaching the central nervous system. Consequently, successful drugs must not only demonstrate favourable activity, metabolic stability, and minimal toxicity but also possess the ability to penetrate the BBB and maintain adequate concentrations in the CNS, posing a significant challenge [171, 172]. Therefore, assessing BBB permeability early in the drug screening process is advisable. Incorporating this factor from the start is vitally important for the eventual clinical usability of the drugs. The complexities introduced by BBB permeability significantly escalate the challenges in developing effective treatments for TDP-43 proteinopathy.

## 5. Conclusion and perspectives

The advancement of diverse drug screening and validation models has markedly enhanced the development of therapies targeting TDP-43 proteinopathy in ALS. Remarkable strides have been made in medical research by integrating cutting-edge technologies and creating novel screening models [6]. Developing models for screening genetic modulators has opened new opportunities for treating the disease [173, 174]. The discovery of various protein kinase pathways and various forms of TDP-43 proteinopathy such as misfolding, hyperphosphorylation, and mislocalization also provides promising targets for the treatment of ALS. Gene-targeted therapies are also proving crucial in decoding the mechanisms underlying TDP-43 proteinopathy and hold significant promise as potent tools in combating this disorder in the future. These therapies and potential treatments have emerged from an extensive array of ALS drug screening and validation models that have been meticulously developed. Although these models present certain limitations, they remain essential and invaluable in the ongoing quest to develop effective ALS treatments.

Several ongoing studies are devising innovative approaches to mitigate the limitations inherent in existing disease models. One such advancement is the utilization of iPSC-MNs, which facilitate the creation of patient-specific cells that possess disease-relevant genetic mutations. This approach transcends the constraints of traditional cellular and animal models [175]. In response to the shortcomings of standard models, there is an increasing shift towards integrating diverse models to establish cross-species platforms. This strategy capitalizes on the unique strengths of each model, thereby enhancing the efficiency of screening processes. For instance, the synergy of high-throughput drug screening techniques with *in vivo* validation models allows for the selection of optimal therapeutics, ensuring both efficacy and precision in drug development. Another promising strategy involves the concomitant use of various small molecules that target multiple facets of TDP-43 pathology, aiming to uncover potential synergistic effects [176]. The future of drug development appears to be leaning towards amalgamating drug combinations with multi-model approaches to assess their protective impacts comprehensively. Overall, the relentless pursuits by scientists globally herald a promising future for treating ALS as these innovative methodologies continue to evolve and integrate.
